# Efficacy of cryotherapy on chemotherapy-induced peripheral neuropathy in patients with breast cancer: a propensity score-matched study

**DOI:** 10.1097/MS9.0000000000000906

**Published:** 2023-05-23

**Authors:** Min Xu, Fan Wang, Xiaoli Zhu, Zhaohua Hao

**Affiliations:** a Breast Surgery Affiliated Cancer Hospital and Institute of Guangzhou Medical University; b Nursing Department Affiliated Cancer Hospital and Institute of Guangzhou Medical University; c Cancer Hospital and Institute of Guangzhou Medical University, Guangzhou, People’s Republic of China

**Keywords:** breast cancer, chemotherapy, cryotherapy, peripheral neuropathy

## Abstract

Chemotherapy-induced peripheral neuropathy (CIPN) is one of the most frequent unfavorable side effects. CIPN refers to the loss of peripheral nerve function that certain types of chemotherapy can cause.

**Objective:**

This study investigated the effectiveness of cryotherapy in preventing CIPN and its effect on the quality of life (QoL) during chemotherapy.

**Methods:**

Eligible participants are cancer patients who began therapy with carboplatin, docetaxel, or paclitaxel in the Breast Oncology Unit between May 2022 and October 2022. Patients were distributed into intervention groups that utilized cryotherapy with ice gloves and ice boots and control groups that did not receive cryotherapy. Patient self-report questionnaires were used to quantify patients’ symptoms and QoL after treatment.

**Results:**

The intervention group exhibited significantly less cold sensitivity, hand and foot numbness, and hand tingling than the control group. Daily CIPN symptoms were substantially milder in the intervention group. Before and after treatment, nerve pain, balance, and muscle and joint discomfort were similar. Intervention and control groups have varied neurotoxicity adverse reaction scores. 2.4% of controls had grade 4 motor neurotoxicity impairment. Physical function and QoL improved in the intervention group.

**Conclusions:**

Cryotherapy relieves CIPN symptoms in breast cancer patients receiving carboplatin and paclitaxel chemotherapy. More thorough trials should be carried out to determine the best time limit and duration of cryotherapy.

## Introduction

Highlight

- Patients who underwent chemotherapy with medicines such as oxaliplatin and paclitaxel were shown to be more likely to suffer from peripheral nerve illnesses, according to the findings of our clinical nursing work.
- We intervened in patients using freezing gloves and freezing socks.
- Cryotherapy has a beneficial therapeutic effect on the prevention of CIPN, which has important repercussions for the patients.

Globally, adjuvant systemic therapy has greatly enhanced breast cancer survival^1^. It has been demonstrated that adjuvant chemotherapy reduces the risk of breast cancer recurrence and mortality^2^. Chemotherapy-induced peripheral neuropathy (CIPN) is one of the most prevalent side effects in chemotherapy patients^3^. The overall incidence of CIPN in cancer patients is more than 60%^4^, and 20–30% of patients are long-term sufferers^5,6^. CIPN is the deterioration of peripheral nerve function caused by specific chemotherapy medications (such as oxaliplatin, taxanes, vinblastines, etc.)^7^.

CIPN may give rise to a range of sensory, motor, and autonomic manifestations. These may include symmetrical numbness, tingling, heightened sensitivity to cold and temperature, and muscle weakness in the extremities^8–10^. Seretny’s Meta-Analysis revealed that oxaliplatin, paclitaxel, and cisplatin/carboplatin combined with paclitaxel caused CIPN in 72.3%, 70.8%, and 73.0% of patients, respectively^9^. In addition, CIPN might manifest minutes after chemotherapy and disappear within 2–3 days^11^, or it can persist decades after chemotherapy has ended^12–14^. The use of freezing gloves and socks reduced the likelihood of docetaxel-induced CIPN by 44%, according to an exploratory trial^15^. Cryotherapy not only inhibits the release of vasodilation chemicals, hence decreasing the sensitivity of pain receptors, but it also decreases muscle spasms by decreasing nerve conduction velocity and muscle excitability^16^. Regional hypothermia therapy can reduce regional perfusion, metabolic rate, and cell chemical activity in order to alleviate chemotherapy-related symptoms^17^. There are many studies showing similar benefits of using cryotherapy for the prevention of CIPN; unfortunately, no consensus exists about the therapeutic value of cryotherapy^18–22^.

In light of the correlation between ethnicity and temperature sensitivity, and the lack of a corresponding report in China, a clinical study was conducted to investigate the effectiveness of cryotherapy, specifically the use of freezing gloves and socks, in mitigating CIPN among breast cancer patients undergoing paclitaxel and platinum-based chemotherapy. And our research hypotheses are that in breast cancer patients taking chemotherapy, cryotherapy has a significant benefit in preventing CIPN.

## Materials and methods

### Study design

This study is a prospective trial undertaken at a Cancer Center of a Medical University. The Institutional Review Board approved this study, and written informed permission was obtained from all participants. This study was performed in line with the principles of the Declaration of Helsinki.

### Participants

All adult patients diagnosed with breast cancer at the Breast Oncology Unit of the Center between May and October 2022 and treated with carboplatin, docetaxel, or paclitaxel are eligible to participate in this study. If the patient has other causes of peripheral neuropathy, such as diabetes, infection, radiotherapy, poisoning, alcoholism, etc., or if they have peripheral sensory abnormalities, aberrant neurological illnesses, or are receiving other neurotoxic medicines, they will be excluded. After verifying that the patients were eligible, the grouping of the patients was carried out using the random number table approach. Patients were randomly randomized to cryotherapy with frozen gloves and socks or a control group that did not get cryotherapy. The patients will be segregated into two distinct nursing cohorts for the purpose of therapy subsequent to group allocation, with the aim of reducing the impact of contamination.

### Sociodemographic and clinical characteristics

The clinical and sociodemographic features of patients were taken from their medical records. Comorbidities were assessed through the use of a self-reported comorbidity questionnaire in addition to an examination by a professional nurse.

### Cryotherapy intervention

The intervention group applied cryotherapy to the hands and feet of patients using cryo gloves and socks. The outside material of frozen gloves and stockings is thermoplastic polyurethane. Additionally, polyacrylic polyol polymer is used inside as a refrigerant for freezing. To get the optimal ideal temperature, place the gloves in a −20°C freezer for at least 3 h before usage. The subjects wore freezing gloves and socks from 15 min prior to chemotherapy infusion to 15 min after chemotherapy infusion and continued to wear the device during treatment. Paclitaxel is typically administered within 30 min; therefore, cryotherapy typically lasts 60 min. Gloves and socks can be kept at a temperature of between −10 and 4°C when in use. At regular intervals of 45 min, the cryostat was substituted to uphold optimal vasoconstrictive cryogenic temperatures. Furthermore, as a measure of hygiene, the participants utilized disposable nitrile gloves and polypropylene sock liners within their gloves and socks, correspondingly. Participants with cryotherapy intolerance were permitted a brief pause during cryotherapy administration.

### Measurements

#### CIPNAT

Tofthagen *et al*.^23^ developed Chemotherapy-Induced Peripheral Neuropathy Assessment Tool (CIPNAT) in 2008 as a patient self-report questionnaire that can completely measure the incidence of CIPN in cancer patients. The scale consists of two parts: the first is the patient’s experience with CIPN symptoms, and the second is the influence of CIPN on the patient’s everyday activities. CIPN symptom experience consists of nine symptoms, which are separated into two categories of sensory and motor nerve injury, with a total of 36 subitems rating symptom occurrence, severity, degree of distress, and frequency of occurrence, yielding a total score of 0–279 points. The higher the score, the more severe the patient’s CIPN symptoms. The influence of CIPN on the quality of life (QoL) of patients is measured along two dimensions – fine motor and general activities – with a total of 14 items yielding a score between 0 and 140. The greater the total score, the greater the daily effect of symptoms. This study employs the Chinese version of the CIPNAT, which preserves all the original items of the original scale and maintains the original scoring procedure and dimension divisions. In the Chinese version, the test–retest reliability of each subscale and the overall scale is between 0.89 and 0.93, the Cronbach’s coefficient is between 0.891 and 0.941, and the content validity index is between 0.89 and 1. Exploratory factor analysis reveals that the factor loading of each item is greater than 0.4 and that the cumulative contribution rate is 57.46%, indicating that the Chinese version of the CIPNAT is reliable and valid.

#### QoL

In this study, the EORTC QLQ-C30 was utilized to assess the QoL of patients. The scale consists of 30 items and 15 domains, comprising 5 functional subscales, 3 symptom subscales, 6 individual measurement items, and 1 domain assessing general health status. In addition to the overall QoL rating, which ranged from 1 (‘extremely poor’) to 7 (‘outstanding’), each item was assessed using a Likert scale ranging from 1 (‘not at all’) to 4 (‘very much’). The scores of each subscale were transformed to a scale ranging from 0 to 100, with higher scores indicating a higher QoL. Wan reported that the Chinese version of EORTC QLQ-C30 was used in our research. Both the internal consistency and test–retest reliability of the Chinese version are more than 0.70, showing that it is reliable, valid, and feasible.

#### The incidence of CIPN

The National Institutes of Health (NIH) and the National Cancer Institute (NCI) completed a revision of the National Cancer Institute Common Terminology Criteria for Adverse Events (NCI-CTCAE 5.0) in 2017. This version is the most recent revision of this document. The grading standard for peripheral neurotoxicity was utilized in this study to analyze the incidence of central and peripheral neuropathy (CIPN) in patients and to rate the adverse reactions that manifested themselves in each system^24^.

### Statistical analysis

Using the PASS software, the sample size can be estimated based on the following assumptions: the predicted dropout rate of the sample is 20%, the significance level =0.05, the test power is 0.80, and the significance level =0.05. As a result, there were a minimum of 45 samples included in each of the groups, in order to make the matching more accurate, the sample size of the control group and the intervention group was set to 2:1; for a total of 135 instances.

IBM SPSS 26.0 was used to conduct all of the statistical analyses. Depending on whether or not the continuous data were regularly distributed, the mean, standard deviation, or interquartile range (IQR) was used to summarize them. If the data were not normally distributed, the median was used. The results of categorical research are often displayed using counts (*N*) and percentages (%). The self-reported outcomes of CIPNAT and QoL in the experimental and control groups before and after chemotherapy were subjected to one-way analysis of variance (ANOVA) testing to determine whether or not they were independent.

In order to correct for any imbalance caused by the use of subjective patient self-report questionnaires, propensity score matching (PSM) will be used in addition to a thorough analysis of all measurement findings. The propensity score for this study was calculated using six predetermined covariates: patient age, sex, tumor metastasis, chemotherapeutic regimen, and CIPNAT, or NCI-CTCAE score baseline. The closest propensity score was utilized in a greedy 1:1 algorithm to pair patients getting cryotherapy with those in the control group for each pair of patients.

## Results

### Sociodemographic and clinical characteristics

**Figure 1:**
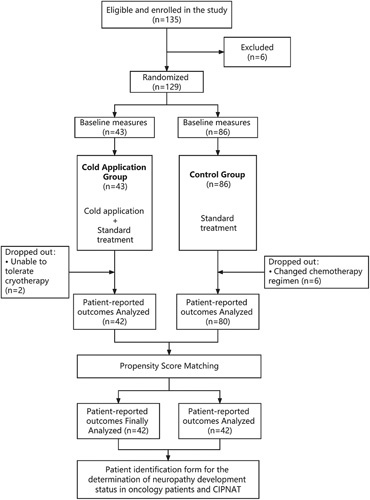
Flowchart.

There were a total of 135 breast cancer patients enrolled in the study. Seven patients withdrew from the study after randomization due to changes in chemotherapy regimen or inability to endure cryotherapy (Fig. 1). The age of 122 breast cancer patients is 49.93±10.74 years old; there were no significant sociodemographic or clinical differences between the intervention groups and control groups. They all received systemic chemotherapy, including nab-paclitaxel, paclitaxel, docetaxel, and carboplatin. The medical information and features of the individuals in the study are presented in Table 1.

**Table 1:** Characteristics of the overall study population and matched cohort, based on whether cryotherapy was administered

	Cryotherapy, *n*=42	Control, *n*=80	*P*	Control matched, *n*=42	*P*
Age, years, mean (SD)	49 (11.4)	49.03 (10.7)	0.004	51.1 (9.8)	0.372
Type of tumor, *n* (%)					
Invasive breast cancer	22 (52.4)	39 (48.8)	0.025	19 (43.9)	0.57
Invasive ductal carcinoma of the breast	17 (40.5)	38 (47.5)		21 (51.2)	
Invasive papillary carcinoma of the breast	0 (0)	1 (1.3)		1 (2.4)	
Infiltrating lobular carcinoma of the breast	2 (4.8)	1 (1.3)		1 (2.4)	
Invasive medullary carcinoma of the breast	1 (2.4)	1 (0)		0 (0)	
Breast cancer stage, *n* (%)					
Stage 1	4 (9.5)	3 (3.8)	0.033	1 (2.4)	0.22
Stage 2	16 (38.1)	28 (35.0)		10 (24.4)	
Stage 3	16 (38.1)	30 (37.5)		17 (41.5)	
Stage 4	3 (7.1)	8 (10.0)		8 (19.5)	
Unstaged	3 (7.1)	11 (13.8)		5 (12.2)	
Cancer histology, *n* (%)					
ER positive	21 (50.0)	45 (56.3)	0.099	24 (58.5)	0.86
PR positive	14 (33.3)	30 (37.5)		16 (39.0)	
HER-2 positive	22 (52.4)	20 (25)		20 (48.8)	
Metastases, *n* (%)					
Lung metastases	5 (11.9)	8 (10.0)	<0.001	3 (7.3)	0.68
Liver metastases	1 (2.4)	0 (0)		0 (0)	
Bone and lymphatic metastasis	3 (7.1)	3 (3.8)		2 (4.9)	
Chest wall metastasis	1 (2.4)	6 (7.5)		2 (4.9)	
Systemic multiple metastases	1 (2.4)	5 (6.3)		3 (7.3)	
Breast lumpectomy, *n* (%)					
Yes	24 (57.1)	48 (60.0)	0.017	22 (46.3)	0.83
No	18 (42.9)	32 (40.0)		19 (46.3)	
Chemotherapy, *n* (%)					
Nab-paclitaxel	22 (52.4)	39 (48.8)	0.20	30 (73.2)	0.23
Paclitaxel liposome	17 (40.5)	22 (27.5)		9 (22.0)	
Carboplatin	14 (33.3)	31 (38.8)		17 (41.5)	
Docetaxel	1 (2.4)	4 (5.0)		1 (2.4)	
Number of cycles received, mean (SD)	3.97 (2.35)	5.51 (3.23)	<0.001	4.06 (2.40)	0.88
Targeted therapy, *n* (%)					
Trastuzumab and pertuzumab	11 (26.2)	30 (37.5)	0.045	13 (31.7)	0.08
Trastuzumab	5 (11.9)	27 (33.8)		0 (0)	
Toripalimab	1 (4.8)	11 (13.8)		3 (7.3)	
Tislelizumab	2 (11.9)	12 (15.0)		4 (9.8)	
Comorbidities, *n* (%)					
Diabetes	2 (4.8)	12 (15.0)	0.005	4 (9.8)	0.61
Peripheral nerve disease	2 (4.8)	7 (8.8)		1 (2.4)	
Osteoarthrosis	2 (4.8)	5 (6.3)		1 (2.4)	
Neurotrophic drug therapy, *n* (%)					
Mecobalamin	0 (0)	7 (8.8)	0.21	3 (7.3)	0.40
Oryzanol	2 (7.1)	3 (3.8)		1 (2.4)	
BMI, kg/m^2^, *n* (%)					
<18.5 (underweight)	1 (2.4)	5 (6.3)	0.57	3 (7.3)	0.65
18.5–24.9 (normal weight)	25 (59.5)	42 (52.5)		25 (60.9)	
25–29.9 (overweight)	13 (31.0)	21 (26.3)		12 (29.3)	
≥30 (obese)	3 (7.1)	12 (15.0)		1 (2.4)	
Marital status, *n* (%)					
Married/living together	41 (97.6)	77 (96.3)	0.008	41 (100)	1.0
Divorced/separated	1 (2.4)	3 (3.8)		0 (0)	
The number of children born, *n* (%)					
1	13 (30.9)	20 (25.0)	0.32	10 (24.4)	0.46
2	19 (45.2)	38 (47.5)		16 (39.0)	
≥3	10 (23.8)	22 (27.5)		15 (36.6)	
Education level, *n* (%)					
Primary school	11 (26.2)	33 (41.3)	0.06	14 (34.2)	0.41
Junior high school	19 (45.2)	25 (31.3)		12 (29.3)	
High school or technical secondary school	6 (14.3)	9 (11.2)		5 (12.2)	
Bachelor’s degree or higher	6 (14.3)	13 (16.3)		10 (24.4)	

ER, estrogen receptor; PR, progesterone receptor.

### PSM

The intervention group and the treatment group’s propensity scores were matched, resulting in 42 subject groups with matched clinical information and baseline peripheral nerve function. These matched patients’ CIPNAT self-report scores and NCI-CTCAE scoring were not significantly different (*P*>0.05). Table 2 contains a list of the precise baseline data. After PSM, baseline data from the intervention and control groups are matched (CIPNAT and NCI-CTCAE)

**Table 2:** Comparison of baseline data between the intervention and control groups following propensity score matching (CIPNAT and NCI-CTCAE)

	Cryotherapy matched	Control matched	*t*	DOF	*P*
Sample size after matching	42	42	—	—	—
CIPNAT Patient Self-report Score					
Neuropathy Interference	18.71	23.03	−0.433	60	0.67
Neuropathy Symptom experience	12.55	11.32	0.235	57.33	0.82
NCI-CTCAE Classification					
Sensory neurotoxicity	0.93	0.89	0.178	53.92	0.86
Motor neurotoxicity	0.71	0.75	−0.166	54	0.87
Neuralgia	0.61	0.46	0.721	54	0.47

CIPNAT, Chemotherapy-Induced Peripheral Neuropathy Assessment Tool; DOF, degrees of freedom; NCI-CTCAE, National Cancer Institute Common Terminology Criteria for Adverse Events.

### CIPN

**Figure 2:**
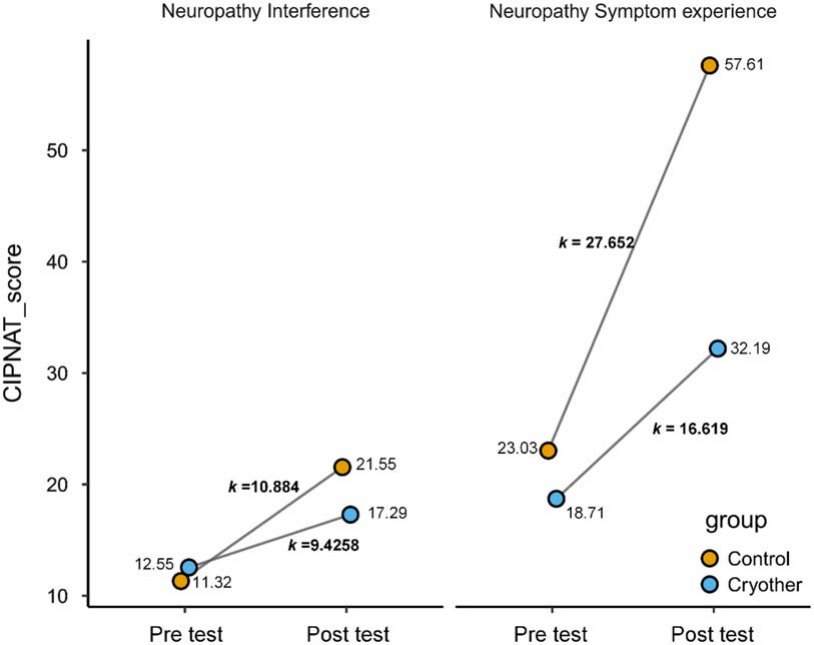
Mean chemotherapy-induced peripheral neuropathy symptom experience and interference scores were compared between the intervention and control groups after propensity score matching.

The CIPNAT self-report questionnaire was used to make comparisons on the prevalence and severity of CIPN among the study participants (Table 3). In terms of symptoms, those in the intervention group experienced less cold sensitivity (*P*=0.022), numb hands (*P*<0.001), numb feet (*P*<0.001), and tingling hands (*P*=0.011) than those in the control group. In all, 90% and 85% of patients in the control groups experienced numbness in their hands and feet, whereas only 54.8% and 42.9% of patients in the intervention group experienced this symptom. After chemotherapy, the intervention group reported lower total symptom scores, indicating that their symptoms were less severe than those reported by the control group. There was not a significant difference between the scores of nerve pain (*P*=0.17), loss of balance (*P*=0.522), foot tingling (*P*=0.537), and muscle and joint pain (*P*=0.488) before and after chemotherapy in the intervention group and the control group. Those results indicated that those in the intervention group were significantly less impacted by the symptoms of CIPN on a day-to-day basis when compared to those in the control group (19.33 vs. 35.22, *P*=0.032). It was discovered that the patients in the control group were more severe and deteriorated than the intervention group, whether in terms of the symptoms of neuropathy or the impact of neuropathy on life after PSM was employed to address the baseline imbalance (Fig. 2).

**Table 3:** Clinical manifestations and severity of peripheral neuropathy in the intervention group and the control group (CIPNAT Patient Self-report Score)

	%		CIPNAT			
	Before chemo	7 days after chemo	Before chemo	7 days after chemo	*F*	*P*
Symptom experience items						
Cold sensitivity						
Cryotherapy	14.3	28.6	2.40 (6.54)	4.17 (7.43)	5.497	0.022
Control	7.5	47.5	1.50 (5.71)	8.05 (9.71)		
Muscle or joint aches						
Cryotherapy	7.3	47.6	0.76 (2.76)	6.57 (8.05)	0.485	0.488
Control	17.5	62.5	2.52 (6.23)	9.70 (9.31)		
Numb hands						
Cryotherapy	31	54.8	4.14 (7.23)	7.04 (8.06)	33.474	<0.001
Control	25	90	3.60 (7.06)	13.40 (8.59)		
Numb feet						
Cryotherapy	21.4	42.9	4.05 (8.43)	6.07 (8.48)	12.244	0.001
Control	25	85	3.33 (6.73)	13.35 (9.42)		
Loss of balance						
Cryotherapy	0	19	0 (0)	2.52 (6.35)	0.413	0.522
Control	12.5	37.5	1.68 (5.69)	5.45 (9.23)		
Muscle weakness						
Cryotherapy	4.8	52.4	0.50 (2.27)	7.31 (8.45)	7.277	0.009
Control	30	62.5	4.08 (7.23)	19.53 (19.01)		
Tingling hands						
Cryotherapy	0	11.9	0 (0)	2.17 (6.08)	6.806	0.011
Control	10	32.5	1.50 (4.99)	6.05 (9.31)		
Tingling feet						
Cryotherapy	4.8	19	0.31 (1.85)	2.29 (5.51)	0.385	0.537
Control	7.5	35	0.80 (3.04)	3.68 (6.20)		
Nerve pain						
Cryotherapy	4.8	59.5	0.31 (1.85)	7.60 (7.98)	1.92	0.170
Control	35	95	4.45 (7.01)	14.5 (7.51)		
Interference items						
Cryotherapy	—	—	8.07 (13.79)	19.33 (24.15)	4.769	0.032
Control	—	—	15.41 (22.29)	40.41 (35.22)		

CIPNAT, Chemotherapy-Induced Peripheral Neuropathy Assessment Tool.

### CIPN symptom burden (NCI-CTCAE)

**Figure 3:**
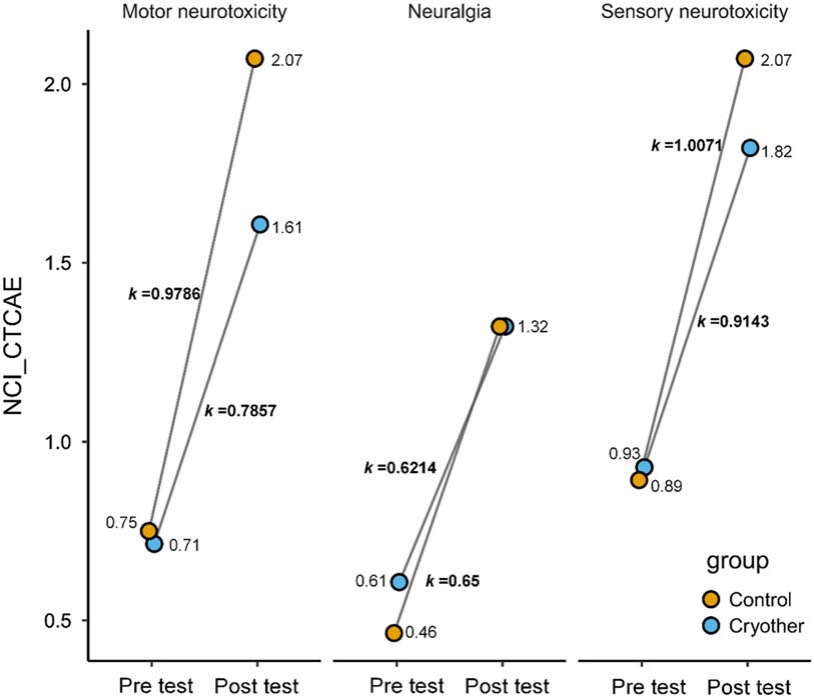
The degree of adverse neurological responses in individuals before and after cryotherapy (NCI-CTCAE – National Cancer Institute Common Terminology Criteria for Adverse Events) between the intervention and control groups after propensity score matching.

Tumor specialist nurses examined and graded each patient’s CIPN symptoms in accordance with NCI-CTCAE criteria on the day before chemotherapy and the seventh day after chemotherapy. The total incidence rate of sensory neurotoxicity before therapy was 61.54%. The values are 48.19% and 38.55% for motor neurotoxicity and neuralgia, respectively (Table 4). Overall, the intervention group’s symptom score stayed below grade 3, and the great majority of patients’ ratings were only rated 1 or 2. As a result, the motor neurotoxicity component in the control group was severely impaired in 2.4% of patients, earning it a grade of 4. After adjusting for baseline differences, motor abnormalities worsened considerably more in the control group than in the intervention group, although sensory abnormalities and neuralgia did not differ significantly (Fig. 3).

**Table 4:** The incidence of neurotoxic adverse reactions before and after chemotherapy in the intervention group and the control group (NCI-CTCAE classification)

	CIPN incidence			
	+	−	*t*	*P*
Sensory neurotoxicity, *n* (%)				
Intervention group				
Pretest	19 (45.24)	23 (54.76)	6.97	<0.001
Posttest	39 (92.86)	3 (7.14)		
Control group				
Pretest	33 (78.05)	9 (21.95)		
Posttest	41 (97.56)	1 (2.44)		
Total				
Pretest	52 (61.45)	32 (38.55)	—	—
Posttest	71 (95.18)	4 (4.82)		
Motor neurotoxicity, *n* (%)				
Intervention group				
Pretest	14 (33.33)	28 (66.67)	6.104	<0.001
Posttest	37 (88.10)	5 (11.90)		
Control group				
Pretest	26 (63.42)	16 (36.59)		
Posttest	36 (87.80)	6 (12.20)		
Total				
Pretest	40 (48.19)	44 (51.81)	—	—
Posttest	78 (93.98)	6 (6.02)		
Neuralgia, *n* (%)				
Intervention group				
Pretest	12 (28.57)	30 (71.43)	4.4	<0.001
Posttest	34 (80.95)	8 (19.05)		
Control group				
Pretest	20 (48.78)	21 (51.22)		
Posttest	40 (97.56)	1 (2.44)		
Total				
Pretest	32 (38.55)	51 (61.45)	—	—
Posttest	74 (89.16)	9 (10.84)		

CIPN, Chemotherapy-induced peripheral neuropathy; NCI-CTCAE, National Cancer Institute Common Terminology Criteria for Adverse Events

### QoL

In order to determine the subjects’ overall QoL, a questionnaire based on the EORTC QLQ-C30 scale was employed for each participant. A comparison of the rise or reduction in their patients’ self-reported QoL scores before and after chemotherapy was made between patients in the intervention group and control group (Table 5). The intervention group did much better in the functional categories. The scores of those in the intervention group were significantly higher than those in the control group in a number of categories, most notably physical function (85.87 vs. 82.76, *P*=0.004) and global QoL (65.08 vs. 50.20, *P*<0.001). Regarding the pain symptoms associated with CIPN, the severity of symptoms was significantly higher in the control group than that in the intervention group (35.77 vs. 14.68, *P*<0.001 for both comparisons). Those results suggested that breast cancer patients undergoing chemotherapy may benefit from the utilization of cryotherapy, particularly in terms of their QoL.

**Table 5:** The quality of life of intervention group and control group before and after cryotherapy (EORTC QoL C30 Patient Self-report Score)

	Cryotherapy		Control			
	Before chemo	7 days after chemo	Before chemo	7 days after chemo	*F*	*P*
Functioning scales						
Physical (PF)	77.14 (16.47)	85.87 (13.75)	87.81 (17.70)	82.76 (14.98)	8.87	0.004
Role (RF)	71.03 (27.06)	80.56 (20.47)	82.11 (24.26)	71.14 (21.42)	14.16	<0.001
Emotional (EF)	80.75 (17.60)	80.16 (19.03)	84.15 (19.79)	68.90 (18.64)	10.17	0.002
Cognitive (CF)	77.38 (20.76)	81.75 (17.96)	86.99 (18.45)	64.23 (21.91)	31.89	<0.001
Social (SF)	62.70 (23.23)	63.10 (21.32)	71.95 (29.45)	54.88 (24.51)	10.04	0.002
Global quality of life (QoL)	54.17 (18.06)	65.08 (20.18)	68.09 (18.90)	50.20 (11.86)	35.83	<0.001
Symptom scales/items						
Fatigue (FA)	30.95 (24.97)	19.58 (20.50)	18.16 (18.55)	47.15 (20.45)	107.32	<0.001
Nausea and vomiting (NV)	13.89 (19.79)	4.76 (10.60)	7.72 (12.96)	30.08 (26.93)	40.62	<0.001
Pain (PA)	32.54 (27.78)	14.68 (19.20)	14.63 (18.71)	35.77 (21.91)	111.06	<0.001
Dyspnea (DY)	15.08 (23.52)	11.11 (20.38)	10.57 (20.33)	21.95 (25.40)	10.00	0.002
Sleep disturbance (SL)	30.95 (29.81)	26.19 (30.83)	20.33 (25.69)	34.96 (27.84)	9.61	0.003
Appetite loss (AP)	40.65 (29.36)	16.26 (25.95)	12.70 (23.23)	27.78 (31.16)	37.73	<0.001
Constipation (CO)	11.11 (19.01)	7.94 (16.15)	12.20 (23.28)	16.00 (28.66)	12.62	0.001
Diarrhea (DI)	13.49 (20.90)	11.11 (19.01)	3.25 (12.48)	13.01 (19.55)	5.98	0.017
Financial impact (FI)	42.86 (32.33	45.24 (28.34)	30.08 (32.32)	46.34 (27.77)	3.99	0.049

EORTC QoL C30, European Organisation for Research and Treatment of Cancer Quality of Life Questionnaire-C30.

## Discussion

Breast cancer patients undergoing chemotherapy may benefit from CIPN prophylaxis with cryotherapy, according to our results in this randomized controlled trial. Not only does it alleviate some symptoms in the hands and feet (such as tingling, numbness, and other similar sensations), but it also improves one’s overall QoL.

It is well known that the pathophysiology of CIPN is quite complex and involves many factors, which is caused by various types of chemotherapy drugs. Patients receiving high doses and multiple courses of therapy were more likely to develop CIPN^25^. Among female breast cancer patients receiving chemotherapy, older age, higher total paclitaxel dose, history of female hormone-related diseases, hypertension, and BMI should all be considered as risk factors for the development of CIPN^26^. Based on the pathogenesis of CIPN, a large number of compounds have been used to prevent or treat CIPN by blocking ion channels, targeting inflammatory cytokines, and resisting oxidative stress^27^. An in-vivo study showed that localized hypothermia reduced sciatic nerve blood flow and neuronal metabolism in rats, implying reduced cumulative doses of toxic chemotherapy near distal nerve fibers^28^.

Worldwide, various studies have confirmed the effect of cryotherapy on the prevention of CIPN. In a Singapore study^29^, cryotherapy individuals showed larger amplitudes of sympathetic skin responses (*β*=0.544, 95% CI=0.108–0.98, *P*=0.014), suggesting a possible autonomic benefit of cryotherapy. However, 80.9% of the subjects temporarily ceased cryotherapy due to cold intolerance. A Japanese study demonstrated that cryotherapy could avoid the objective and subjective symptoms of CIPN and the resultant dysfunction study of 40 patients^18^. A Danish study found that patients wearing freeze gloves and socks/socks had less neuropathy during docetaxel treatment than controls^15^.

In addition, the findings of certain studies have been negative. According to the results of a research project conducted in the United States, there did not appear to be any significant difference between cryotherapy and compression therapy in terms of the frequency of CPIN^30^. Physical activity was found to be more helpful than the application of cold compresses in a trial conducted in Turkey including 90 patients^31^. The researchers found that exercise considerably reduced the CIPN symptoms of hand and foot numbness (*P*=0.009). When comparing patients in Canada who were given cryotherapy to patients who were given a placebo control, researchers in Canada observed no significant difference in EORTC QLQ CIPN-20 symptom scores at any time point between the two groups^32^.

According to our research findings, cryotherapy was beneficial for breast cancer patients receiving chemotherapy medicines. Patients diagnosed with breast cancer who took part in the study and got cryotherapy had, on average, fewer symptoms associated with CIPN following chemotherapy than the patients in the control group did. In terms of QoL, the functional status and QoL of patients in the control group were limited to varying degrees due to the probable influence of CIPN symptoms. On the other hand, this did not occur in the intervention group. On the other hand, the QoL of those who participated in the intervention was either largely preserved or slightly improved. In addition, our research found no instances in which cryotherapy had to be stopped because the patient was unable to tolerate the cold. These observations may be impacted by the length of time spent cooling, the temperature, or the method, all of which call for further investigation. In comparison to the findings of previous researchers, our team found that cryotherapy only needed to be administered for a shorter amount of time overall. The use of freezing gloves and freezing socks was begun about 15 min prior to chemotherapy and continued for the full 30 min following treatment; this may be a significant influence in enhancing patient tolerance.

This study also has some limitations. Patients in the intervention group may be more motivated to fill out the questionnaire because they have high hopes that the treatment will be neuroprotective and help them experience a reduction in the symptoms associated with CIPN. It is possible that patients who turned down the opportunity to take part in the trial were unaware of the selection bias that occurred. The symptoms of CIPN are subjective, and in order to evaluate them, we used patient questionnaires in which they described their symptoms, as well as concurrent examinations of their symptoms by nurses. However, these evaluations are the product of the patient or nurse making a subjective assessment, even though it is based on the patient’s actual experience of the symptoms. It is possible that this affected the findings of the study. We are unable to perform an objective assessment of the patient’s neurological function via objective markers such as electrophysiology due to technical limitations. This is the most significant shortcoming of the study.

## Conclusion

The present investigation aimed to assess the potential of cryotherapy, specifically utilizing ice gloves and boots, in mitigating CIPN symptoms among female breast cancer patients. The implementation of cryotherapy has demonstrated an enhancement in the symptoms of CIPN and the overall QoL in patients undergoing chemotherapy treatment with carboplatin and paclitaxel.

## Ethical approval

This study has been approved by the Institutional Ethics Committee. Institution Name: Medical Ethics Committee, Affiliated Cancer Hospital of Guangzhou Medical University, Guangzhou, China; No.: A2021381, date: 23 November 2020.

## Consent

Written informed consent was obtained from the patient for the publication of this case report and accompanying images. A copy of the written consent is available for review by the Editor-in-Chief of this journal on request.

## Sources of funding

The authors declare that no funds, grants, or other support were received during the preparation of this manuscript.

## Author contribution

M.X.: conceptualization, data curation, methodology, and project administration; F.W.: data curation, project administration, resources, writing – original draft, and writing – review and editing; M.X. and F.W.: have the same contribution to this study; X.Z.: methodology and resources; Z.H.: software, validation, and visualization. All authors read and approved the final manuscript.

## Conflicts of interest disclosure

All authors declare that they have no conflict of interest.

## Research registration unique identifying number (UIN)

1. Name of the registry: Medical Research Project of Guangdong Province.
2. Unique identifying number or registration ID: 2020112015017266.
3. Hyperlink to your specific registration (must be publicly accessible and will be checked): http://kj.gdmde.net/

## Guarantor

Min Xu, BSN, RN, Breast Surgery Affiliated Cancer Hospital and Institute of Guangzhou Medical University, No. 78 Hengzhigang Road, Guangzhou 510030, Guangdong Province, China, E-mail: 2218819834@qq.com

## Provenance and peer review

Our paper has not been invited.

## Data availability statement

The original data for the study may be obtained by contacting the corresponding author when warranted.

## References

[1] Winters S Martin C Murphy D . Breast cancer epidemiology, prevention, and screening. Prog Mol Biol Transl Sci 2017;151:1–32. 29096890 10.1016/bs.pmbts.2017.07.002

[2] Peto R Davies C Godwin J . Comparisons between different polychemotherapy regimens for early breast cancer: meta-analyses of long-term outcome among 100,000 women in 123 randomised trials. Lancet 2012;379:432–44. 22152853 10.1016/S0140-6736(11)61625-5 PMC3273723

[3] Kanzawa-Lee GA . Chemotherapy-induced peripheral europathy: nursing implications. J Infus Nurs 2020;43:155–66. 32287170 10.1097/NAN.0000000000000368

[4] Simon NB Danso MA Alberico TA . The prevalence and pattern of chemotherapy-induced peripheral neuropathy among women with breast cancer receiving care in a large community oncology practice. Qual Life Res 2017;26:2763–72. 28664460 10.1007/s11136-017-1635-0

[5] Molassiotis A Cheng HL Lopez V . Are we mis-estimating chemotherapy-induced peripheral neuropathy? Analysis of assessment methodologies from a prospective, multinational, longitudinal cohort study of patients receiving neurotoxic chemotherapy. BMC Cancer 2019;19:132. 30736741 10.1186/s12885-019-5302-4 PMC6368751

[6] Ma J Kavelaars A Dougherty PM . Beyond symptomatic relief for chemotherapy-induced peripheral neuropathy: targeting the source. Cancer 2018;124:2289–98. 29461625 10.1002/cncr.31248 PMC5991994

[7] Zajączkowska R Kocot-Kępska M Leppert W . Mechanisms of chemotherapy-induced peripheral neuropathy. Int J Mol Sci 2019;20:1451. 30909387 10.3390/ijms20061451 PMC6471666

[8] Trecarichi A Flatters SJL . Mitochondrial dysfunction in the pathogenesis of chemotherapy-induced peripheral neuropathy. Int Rev Neurobiol 2019;145:83–126. 31208528 10.1016/bs.irn.2019.05.001

[9] Seretny M Currie GL Sena ES . Incidence, prevalence, and predictors of chemotherapy-induced peripheral neuropathy: a systematic review and meta-analysis. Pain 2014;155:2461–70. 25261162 10.1016/j.pain.2014.09.020

[10] Staff NP Grisold A Grisold W . Chemotherapy-induced peripheral neuropathy: a current review. Ann Neurol 2017;81:772–81. 28486769 10.1002/ana.24951 PMC5656281

[11] Wang YJ Chan YN Jheng YW . Chemotherapy-induced peripheral neuropathy in newly diagnosed breast cancer survivors treated with taxane: a prospective longitudinal study. Support Care Cancer 2021;29:2959–71. 33025227 10.1007/s00520-020-05796-0

[12] Mols F Beijers T Lemmens V . Chemotherapy-induced neuropathy and its association with quality of life among 2- to 11-year colorectal cancer survivors: results from the population-based PROFILES registry. J Clin Oncol 2013;31:2699–707. 23775951 10.1200/JCO.2013.49.1514

[13] Park SB Goldstein D Krishnan AV . Chemotherapy-induced peripheral neurotoxicity: a critical analysis. CA Cancer J Clin 2013;63:419–37. 24590861 10.3322/caac.21204

[14] Storey S Cohee A Von Ah D . Presence and distress of chemotherapy-induced peripheral neuropathy symptoms in upper extremities of younger and older breast cancer survivors. J Patient Cent Res Rev 2020;7:295–303. 33163549 10.17294/2330-0698.1757 PMC7644124

[15] Eckhoff L Knoop AS Jensen MB . Risk of docetaxel-induced peripheral neuropathy among 1,725 Danish patients with early stage breast cancer. Breast Cancer Res Treat 2013;142:109–18. 24132874 10.1007/s10549-013-2728-2

[16] Kokotis K . Preventing chemical phlebitis. Nursing 1998;28:41–6. 10.1097/00152193-199811000-00021 9856034

[17] Kadakia KC Rozell SA Butala AA . Supportive cryotherapy: a review from head to toe. J Pain Symptom Manage 2014;47:1100–15. 24210702 10.1016/j.jpainsymman.2013.07.014 PMC4013268

[18] Hanai A Ishiguro H Sozu T . Effects of cryotherapy on objective and subjective symptoms of paclitaxel-induced neuropathy: prospective self-controlled trial. J Natl Cancer Inst 2018;110:141–8. 29924336 10.1093/jnci/djx178 PMC6007752

[19] Loprinzi CL Lustberg MB Hershman DL . Chemotherapy-induced peripheral neuropathy: ice, compression, both, or neither. Ann Oncol 2020;31:5–6. 31912795 10.1016/j.annonc.2019.10.009

[20] Scotté F Banu E Medioni J . Matched case–control phase 2 study to evaluate the use of a frozen sock to prevent docetaxel-induced onycholysis and cutaneous toxicity of the foot. Cancer 2008;112:1625–31. 18286527 10.1002/cncr.23333

[21] van den Hurk CJ Peerbooms M van de Poll-Franse LV . Scalp cooling for hair preservation and associated characteristics in 1411 chemotherapy patients – results of the Dutch Scalp Cooling Registry. Acta Oncol 2012;51:497–504. 22304489 10.3109/0284186X.2012.658966

[22] Nangia J Wang T Osborne C . Effect of a scalp cooling device on alopecia in women undergoing chemotherapy for breast cancer: the SCALP randomized clinical trial. JAMA 2017;317:596–605. 28196254 10.1001/jama.2016.20939

[23] Tofthagen CS McMillan SC Kip KE . Development and psychometric evaluation of the chemotherapy-induced peripheral neuropathy assessment tool. Cancer Nurs 2011;34:10–20. 10.1097/NCC.0b013e31820251de 21242773

[24] Tan AC McCrary JM Park SB . Chemotherapy-induced peripheral neuropathy-patient-reported outcomes compared with NCI-CTCAE grade. Support Care Cancer 2019;27:4771–7. 30972648 10.1007/s00520-019-04781-6

[25] Desforges AD Hebert CM Spence AL . Treatment and diagnosis of chemotherapy-induced peripheral neuropathy: an update. Biomed Pharmacother 2022;147:112671. 35104697 10.1016/j.biopha.2022.112671 PMC11118018

[26] Hiramoto S Asano H Miyamoto T . Risk factors and pharmacotherapy for chemotherapy-induced peripheral neuropathy in paclitaxel-treated female cancer survivors: a retrospective study in Japan. PLoS One 2021;16:0261473. 10.1371/journal.pone.0261473 PMC8719717 34972132

[27] Hu LY Mi WL Wu GC . Prevention and treatment for chemotherapy-induced peripheral neuropathy: therapies based on CIPN mechanisms. Curr Neuropharmacol 2019;17:184–96. 28925884 10.2174/1570159X15666170915143217 PMC6343206

[28] Liao LD Orellana J Liu YH . Imaging of temperature dependent hemodynamics in the rat sciatic nerve by functional photoacoustic microscopy. Biomed Eng Online 2013;12:120. 24245952 10.1186/1475-925X-12-120 PMC4225521

[29] Ng DQ Tan CJ Soh BC . Impact of cryotherapy on sensory, motor, and autonomic neuropathy in breast cancer patients receiving paclitaxel: a randomized, controlled trial. Front Neurol 2020;11:604688. 33424755 10.3389/fneur.2020.604688 PMC7793726

[30] Kanbayashi Y Sakaguchi K Ishikawa T . Comparison of the efficacy of cryotherapy and compression therapy for preventing nanoparticle albumin-bound paclitaxel-induced peripheral neuropathy: a prospective self-controlled trial. Breast 2020;49:219–24. 31901783 10.1016/j.breast.2019.12.011 PMC7375545

[31] Simsek NY Demir A . Cold application and exercise on development of peripheral neuropathy during taxane chemotherapy in breast cancer patients: a randomized controlled trial. Asia Pac J Oncol Nurs 2021;8:255–66. 33850959 10.4103/apjon.apjon-2075 PMC8030600

[32] Bland KA Kirkham AA Bovard J . Effect of exercise on taxane chemotherapy-induced peripheral neuropathy in women with breast cancer: a randomized controlled trial. Clin Breast Cancer 2019;19:411–22. 31601479 10.1016/j.clbc.2019.05.013

